# From the Scientific Underground to the Height of Recognition: The 40th Anniversary of Rita Levi-Montalcini’s Nobel Prize and the Promising Horizon in Psychiatry

**DOI:** 10.1007/s11064-026-04769-9

**Published:** 2026-05-26

**Authors:** Larissa Junkes, Antonio E. Nardi, Emilio Clementi

**Affiliations:** 1 Institute of Psychiatry (IPUB), Federal University of Rio de Janeiro (UFRJ), Venceslau Braz Avenue, 71, Botafogo, Rio de Janeiro, 22290-140 Brazil; 2 Department of Biomedical and Clinical Sciences (DIBIC), ASST Fatebenefratelli-Sacco Hospital, Università degli Studi di Milano, Milano, Italy

**Keywords:** Nerve growth factor, Receptors, Intercellular signaling peptides and proteins, Neurobiology, Neuropsychiatry, Depression

## Abstract

Forty years ago, in 1986, Rita Levi-Montalcini was awarded the Nobel Prize in Physiology or Medicine for the discovery of Nerve Growth Factor, a fundamental finding that inaugurated the field of trophic signaling in the nervous system. Her research, conducted with credibility and consistency under the precarious conditions of an Italy enduring the Second World War, and later refined in the United States, rigorously demonstrated that neuronal growth and survival are regulated by specific chemical signals, not merely by intrinsic processes. Using biological assays involving sarcoma and chicken embryos, Levi-Montalcini identified a protein substance that promoted exuberant nerve fiber growth from sensory and sympathetic ganglia. The subsequent purification and biochemical characterization of Nerve Growth Factor by biochemist Stanley Cohen — with whom she shared the Nobel Prize — confirmed its protein nature. This discovery scientifically proved the existence of signaling molecules that guide nervous system development, including critical processes such as neuronal differentiation, axonal growth, and apoptosis, programmed cell death. The concept that target-cells secrete trophic factors for maintenance and survival became fundamental in neuroembryology. The impact of Levi-Montalcini’s work transcended developmental neurobiology. The Nerve Growth Factor model paved the way for the discovery of a family of neurotrophins, elucidating mechanisms of neural plasticity, regeneration, and brain aging, with profound implications for the understanding of mental illnesses, neurodegenerative diseases, and cancer. For establishing, with experimental precision, a universal principle of cellular communication in the nervous system, Rita Levi-Montalcini — a scientist who expanded scientific frontiers — was honored with numerous awards, alongside the Nobel Prize, consolidating her position as an influential female figure in 20th-century neuroscience.

## Overlapping Temporalities: Sustained Research Amid the Scourges of War

Rita Levi-Montalcini (1909–2012) was born on April 22 into a family of Jewish origin [1] in the Italian city of Turin [2]. The daughter of painter Adele Montalcini and mathematician and electrical engineer Adamo Levi, she had three siblings: Gino, the eldest, became an architect and professor at the University of Turin; and two sisters, Anna, five years older, and Paola, her twin sister, together with Rita the youngest daughters of the couple. Anna was an admirer of the Swedish writer and Nobel Prize in Literature winner of 1909 — the year of Rita’s birth — Selma Lagerlöf, and instilled in her such enthusiasm that Rita even considered pursuing a career in literature and “describing an Italian saga ‘à la Lagerlöf’” [3]. Her father did not encourage his daughters to continue their studies, fearing that a professional life might hinder the natural expectation of the time, repeatedly confined to the home as wives and mothers. However, he supported his daughter Rita when she chose to study Medicine at the University of Turin [4], a decision made after the death of a family friend from stomach cancer.

**Fig. 1:**
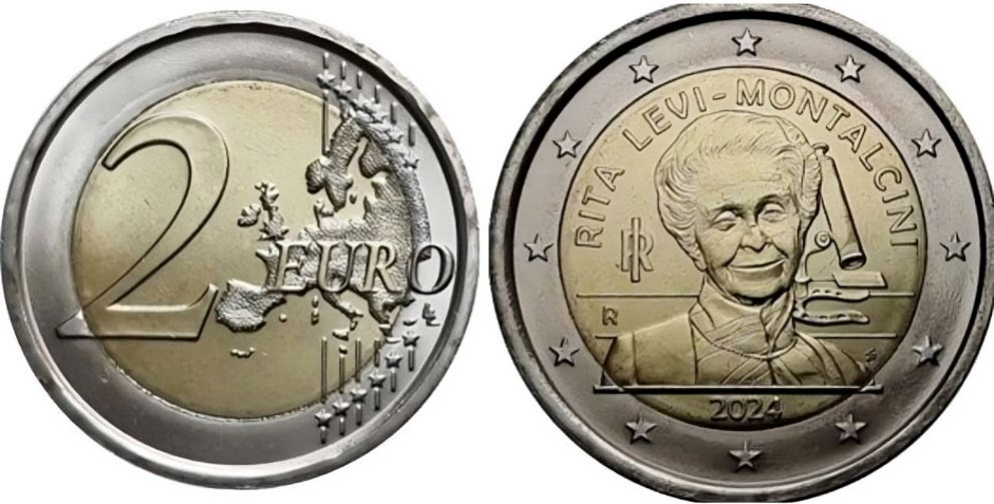
Commemorative two-euro coin of Rita Levi-Montalcini, issued by Italy as part of a series called ‘Donne nella Storia d’Italia’ (Women in the History of Italy), which honors notable female figures

Rita graduated in Medicine in 1936 — and maintained a close connection with colleagues Renato Dulbecco and Salvador Luria [5] — remaining in the anatomy department at the same University of Turin as an assistant to her Neurology Professor, Giuseppe Levi. Subsequently, in the Kingdom of Italy under fascism, in 1938, her academic career was interrupted by Benito Mussolini’s Racial Manifesto, with anti-Semitic laws imposing marginalization and discrimination, resulting in the loss of protective structures that deprived Jews of Italian citizenship and governmental and professional positions [6]. Removed from the university and aiming to continue her research, she adapted a home laboratory in her bedroom to resume her study on how the periphery affected the development of the nervous system in its early stages. The object of the experiments were chicken embryos — she would bicycle to neighboring farms to purchase fertilized chicken eggs — which she incubated at home using a small thermostat [7] (Fig. 1).

During that period, she observed for the first time the occurrence of massive cell death, even in normally developing embryos [8]. Although the term ‘apoptosis’ was coined in 1972, it was Levi-Montalcini, together with Giuseppe Levi, who originally described a spontaneous form of neuronal death in chicken embryos in the 1940s [9], in an article of limited reach, printed by a religious institution, as a consequence of Mussolini’s segregationist law and the unusual abstract, in Latin, “*Extirpato*,* ex pulli gallinacei fetu*,* tertio incubationis die”*.

With pessimism of the intellect and optimism of the will [10], all the research secretly conducted by Levi-Montalcini under the totalitarian regime was carried out without any financial or institutional support, given that she was forbidden from publishing articles in Italian scientific journals due to the discriminatory policies of the war [7]. How to endure such pressures and remain sensitive? In a testament to resilience, attempting to offer words for the unspeakable, in an interview given in 2008, she observed that such obstacles did not concern her, and that research with chicken embryos sustained and energized her during those troubled times [5].

Amid fragmentation, a sense of disorientation, and the violence of the unstable period of the German occupation of Italy — which occurred after the Italian surrender to the Allies in September 1943 — the Levi-Montalcini family took refuge in Florence, where the young Rita set up another home laboratory. During that period, she volunteered in the medical service of the Allied Forces until the family returned to Turin in 1945. In September 1946, she received a one-semester fellowship at the laboratory of Professor Viktor Hamburger at Washington University in St. Louis, Missouri, in the United States, where she remained as a collaborator for over 20 years. There, in 1952, she conducted her most important work: the isolation of Nerve Growth Factor (NGF), through the observation of tumor tissues that caused rapid growth of nerve cells. In 1958, Levi-Montalcini became a full professor, and in 1962, she established a laboratory in Rome, dividing her time with her work in Missouri. From 1961 to 1969, she directed the Neurobiological Research Center of the National Research Council in Rome and, from 1969 to 1978, led the Cell Biology Laboratory [3]. After her retirement in 1977, she was appointed director of the Institute of Cell Biology of the National Research Council of Italy, also based in Rome, from which she retired in 1979, continuing as a visiting professor [11]. To further develop NGF-based therapies and attract scientists, she founded the European Brain Research Institute (EBRI), also in Rome, in 2002, with studies covering neurodegenerative diseases [12] (Fig. 1).

The trajectory of Rita Levi-Montalcini mirrors a microcosm of the complex negotiations between scientific knowledge, power structures, and the capacity to innovate: the power of the State and ideology shaping the scientific environment, subjugating science under authoritarianism — whether to promote desirable innovations, such as the electroconvulsive therapy of Ugo Cerletti and Lucio Bini [13]— or to persecute those who did not fit in, like Levi-Montalcini, who maintained science as a refuge in a trajectory where the State was the tormentor. The progression and validation of her discovery occurred only when she was legitimately received by another renowned institution, Washington University in St. Louis, with a model of open science opposed to the closed one promoted by fascism. Her work is a testament to how institutional power can both attempt to annihilate and leverage talent; and to the importance of looking at individual ‘geniuses’ without forgetting the essential support networks, the other researchers, the university and hospital institutions, funding bodies, and the sociopolitical context of each era.

## From Experimental Neurogenesis to Malignant Tissues Elucidating the Enigma

In the 1940s, interest in experimental neuroembryology was declining [14]. In 1934, when Viktor Hamburger replaced the chicken embryo with the amphibian larval embryo as the preferred object for analyzing the effects of limb bud extirpation on spinal motor neurons and sensory nerve cells innervating the limbs [15], a series of investigations began aimed at analyzing this and other related experimental systems in avian embryos. In-depth studies of the chicken embryo nervous system using the silver techniques of Ramon y Cajal and collaborators [16], refined by Levi-Montalcini and colleagues, enabled a more accurate understanding of various nerve centers and their developmental history during neurogenesis, allowing the detection of even small deviations from the rules of normal development in experimentally manipulated embryos — since in chicken embryos, processes occur according to a rigidly programmed temporal sequence, enabling comparison of central and peripheral nerve centers of experimental and control species in embryos incubated under the same environmental conditions [14]. In partnership, Levi-Montalcini and Hamburger conducted research through serial studies of silver-stained embryos, finding that severe hypoplasia of nerve centers deprived of their innervation fields resulted from the death of differentiated neurons, and not from failure to recruit neurons from a pool of still-uncommitted nerve cell precursors [17]. In 1947, at Hamburger’s invitation, Levi-Montalcini left Italy for Washington University to reinvestigate the question, beginning a period of over 20 years of partnership and eternal friendship between the researchers, resulting in the confirmation of the jointly published hypothesis [18]. In the United States, she maintained her friendship with Salvador Luria and Renato Dulbecco, who had moved around the same time — Luria having fled Europe after the German army invaded Paris, and Dulbecco having boarded the same ship bound for New York, together with Levi-Montalcini [5].

In 1943, the scientist Elmer Bucker, a former pupil of Hamburger, reported results from an experiment in which he grafted fragments of mouse ‘sarcoma 180’ onto the body wall of three-day-old chicken embryos, demonstrating that the tumor selectively attracted sensory nerve fibers, increasing the volume of the corresponding ganglia [19]. Levi-Montalcini and Hamburger confirmed and expanded this discovery, revealing that the tumor also attracted sympathetic fibers, which formed a dense network without synaptic connections [20]. Subsequent experiments showed another departure from the norm: in embryos with transplanted mouse sarcoma 180, embryonic viscera that in normal species are devoid of innervation were filled with sympathetic nerve fibers at early embryonic stages [21].

The research in Rio de Janeiro was decisive in confirming the diffusible nature of the growth-promoting activity of sarcoma tumors implanted in the chorioallantoic membrane [21, 22]. Levi-Montalcini, facing technical limitations at Washington University, conducted essential experiments at the Institute of Biophysics of the University of Brazil (now the Federal University of Rio de Janeiro), under the welcoming support of Professor Carlos Chagas, and where her friend Hertha Meyer worked. The tissue culture technique, applied to mouse sarcoma transplants, demonstrated that a soluble agent released by neoplastic cells was responsible for the generalized hyperplasia of the sympathetic chain and the formation of intravascular neuromas, independent of direct contact between tissues. Levi-Montalcini highlighted that “the tumor had given its first hint of its existence in St. Louis, but it was in Rio de Janeiro that it revealed itself, and it did so in a theatrical and grandiose manner, as if stimulated by the brilliant atmosphere of that explosive and exuberant manifestation of life that is Carnival in Rio” [23].

The activity of humoral factors was demonstrated by the growth response to a soluble tumor agent, identified through in vitro bioassay, exhibiting a halo of nerve fibers of maximum density on the side facing the tumor [24]. The isolation of an active nucleoprotein fraction from tumors by Stanley Cohen, [25] preceded the accidental discovery of the potent neurotrophic activity of snake venom, an activity also found in the submandibular gland of mice. The injection of the purified protein into embryos caused massive hypertrophy of sensory and sympathetic ganglia and anomalous innervation of organs, confirming the central role of this molecule in neurogenesis [26].

## The Potentialities of NGF as a Retrograde Trophic Messenger and as a Tropic Factor

The essential role of NGF in early differentiation was demonstrated by immunosympathectomy: administration of anti-NGF antiserum to neonatal rodents eliminated the sympathetic system without compromising general development [27]. In vitro evidence for the role of NGF in the early developmental stages of sensory nerve cells was confirmed in subsequent tests that proved that administration of antiserum against NGF to rodent fetuses [28, 29] and autoimmunization of pregnant rodents against endogenous NGF result in the inability of sensory ganglia to undergo normal development [30]. Immunosympathectomy by anti-NGF antiserum [31] suggested the removal of endogenous circulating NGF as the mechanism, raising questions about its origin and transport to target-cells.

Evidence demonstrated that exogenous NGF prevents neuronal death [32, 33], confirming its vital trophic role. It was further verified that labeled NGF was taken up by nerve terminals and retrogradely transported to cell bodies, supporting the retrograde trophic messenger hypothesis. While its functions in neuronal development and retrograde transport were being consolidated, the fundamental property of NGF’s ability to guide growing or regenerating axons along a concentration gradient (neurotropism) was established, proven by in vivo and in vitro approaches [34, 35]. In vitro studies provided evidence that neurites follow and redirect their growth according to spatial NGF gradients, demonstrating that the neurotropic effect is independent of trophic action. Targets of NGF action include neural crest-derived cells, CNS neurons, and non-neuronal populations, with maximum responsiveness during early differentiation, progressively declining in adulthood, but without complete elimination [36]. NGF’s main activities include the vital trophic action in early development, the promotion of differential processes — such as neurite outgrowth — and the directional guidance of growing or regenerating neurites along a concentration gradient [37].

## Advances in NGF Research in Psychiatry and Depression

Research continues to explore the potential use of NGF in mental illnesses, neurodegenerative diseases such as Alzheimer’s, schizophrenia, and neuropathies, as well as cancer [36, 38]. Clinical trials with NGF-based therapies are advancing via gene therapy and mimetic peptides [39, 40]. The discovery that NGF acts on the immune system, in mast cells and lymphocytes, generated a field of its own, where studies have delved into how NGF regulates inflammation, asthma, autoimmune diseases, and chronic pain [41, 42]. In the 1990s, Levi-Montalcini was one of the first scientists to highlight the importance of mast cells in human pathology [43] and to identify the endogenous compound palmitoylethanolamide as an important modulator of this cell [44]. Understanding this mechanism led to a better understanding of the endocannabinoid system.

The discovery of NGF revolutionized neurobiology by demonstrating that the survival, development, and plasticity of neurons are controlled by specific chemical signals — a revelation that opened a new chapter in the understanding of mental disorders, shifting the exclusive focus from neurotransmitters to the mechanisms that ensure the structural integrity and adaptability of the brain. NGF and the family of neurotrophins it originated, such as brain-derived neurotrophic factor (BDNF) — an essential protein produced by the brain and other tissues, the only neurotrophin related to the intestinal microbiota [45], acts as a “fertilizer” for neurons, promoting their survival, growth, and maintenance, having an important connection with contemporary Psychiatry. BDNF has a regulatory effect on neurogenesis and synaptic plasticity in the hippocampus and prefrontal cortex [46].

The neurotrophic hypothesis of depression [47, 48] proposes that chronic stress and depression are associated with a reduction in neurotrophins such as NGF, vascular endothelial growth factor (VEGF) [49] and, mainly, BDNF, leading to neuronal atrophy and reduced neurogenesis in brain areas such as the hippocampus and prefrontal cortex, with the therapeutic implication being the delay of weeks for the effect of antidepressants, which do not act solely by increasing serotonin but, in the long term, by raising neurotrophin levels, repairing structural damage caused by stress, and restoring neuronal plasticity [50]. Antidepressants such as Selective Serotonin Reuptake Inhibitors (SSRIs), Tricyclic Antidepressants (TCAs), and Monoamine Oxidase Inhibitors (MAOIs) have been shown to significantly increase rates of adult hippocampal neurogenesis through an increase in the number and maturation of neural progenitor cells [51–53]. Antidepressant efficacy in patients with Major Depressive Disorder (MDD) may be associated with the BDNF Val66Met polymorphism [46]. However, the precise role of neurogenesis in depression remains a research gap [54, 55]. There is a potential therapeutic role targeting neurogenesis in MDD, offering a promising pathway for neurogenesis in depression through BDNF regulation, including research pointing to BDNF not only as a possible diagnostic biomarker for depression but as a potential biomarker for monitoring treatment response [56].

Neurotrophins may represent a non-specific vulnerability factor for psychiatric illnesses. An individual’s basal levels of NGF and BDNF, determined by genetics and early life experience, may create a vulnerability gradient, with very low levels hindering the brain’s ability to adapt (plasticity) and recover from insults (stress, trauma), increasing the risk for multiple disorders. The discovery of NGF by Rita Levi-Montalcini transcended developmental biology, providing psychiatry with another paradigm in the evaluation of mental illnesses, not merely as chemical imbalances, but as a failure in the maintenance and plasticity of brain structures, because even the adult brain remains dynamic, requiring constant trophic support to stay healthy.

Neuropsychiatric illnesses such as MDD, schizophrenia, and bipolar disorder alter the generation of new neurons in the adult human brain, disrupting the early stages of hippocampal neurogenesis and its supportive environment, with lifestyle factors further modulating these effects — even in healthy individuals [57]. In patients with depression, significantly smaller hippocampal volumes have been observed using magnetic resonance imaging (MRI) [58, 59]. Regulation of neurogenesis in MDD occurs through the BDNF signaling pathway via its tropomyosin-related kinase B (TrkB) receptor, with preclinical and clinical studies indicating impaired signaling [60]. There is increased phosphorylation of TrkB receptors with most antidepressant medications, which can enhance TrkB signaling and, conversely, transgenic overexpression of TrkB in the brain mimics the effects of antidepressant drugs [61]. Alongside evidence that the hippocampal BDNF-TrkB signaling pathway is involved in the mechanism of action of antidepressants, studies have verified the involvement of nuclear factor kappa B (NF-κB) in the regulation of learning, memory, neurogenesis, survival, and neuronal death in the adult CNS [62], with activation of this NF-κB pathway potentially being necessary to induce neurogenesis and neuronal plasticity in depression [55]. Other signaling pathways, such as Wnt [63] and Toll-like receptors (TLRs) [64] may also be associated with the regulation of neurogenesis in MDD.

Rapid-acting antidepressants — among them ketamine, an N-methyl-D-aspartate receptor (NMDA-R) antagonist — and BDNF and TrkB signaling play a relevant role in this context [60]. A study explored whether antidepressant-like effects of the ketamine enantiomers, arketamine (R-ketamine) or esketamine (S-ketamine), are sustained by protection against cytokine-induced reductions (interleukin-1beta [IL-1β] or IL-6) in hippocampal neurogenesis and by activation of the neurotoxic kynurenine pathway in an in vitro model of depression [65]. The results suggested that R-ketamine and S-ketamine have pro-neurogenic and anti-inflammatory properties, however mediated by inhibition of the kynurenine pathway only in the context of IL-1β. A systematic review of animal models of depression on the shared effects of electroconvulsive therapy (ECT) and ketamine on neuroplasticity showed that hippocampal neurogenesis and BDNF levels increase after ECT and ketamine, with both interventions positively affecting glutamatergic neurotransmission, astrocyte and neuronal morphology, synaptic density, vascularization, and functional plasticity [66]. Rodent studies have revealed that ketamine rapidly increases the release and/or expression of BDNF and VEGF in the prefrontal cortex and hippocampus, which increases the number and function of spine synapses in the prefrontal cortex and hippocampal neurogenesis [49]. More studies are needed to elucidate the complex relationship between BDNF and MDD. Although the relationship between BDNF and depression has been extensively studied, the direct link between serum BDNF levels and brain BDNF and neuroplasticity is not yet well defined.

Several neuropsychiatric and neurodegenerative conditions involve hippocampal dysfunction. Evidence suggests that dorsoventral differences in adult neurogenesis may contribute to different cognitive and affective profiles, where disorders with predominant cognitive impairments involve dysfunction of dorsal neurogenesis, and disorders with emotional, motivational, or anxiety dysregulation are more associated with ventral deficits [53], as in mood disorders.

With the Covid-19 pandemic, research has expanded our knowledge of NGF, as the virus can affect neurotrophic pathways [67, 68]. Recent studies on eye diseases include neurotrophins in the treatment of retinopathy [69]. Developmental neuroscience continues to build upon Levi-Montalcini’s work on axonal guidance and neuronal survival. Levi-Montalcini’s insistence that the reductionist approach should be complemented by a broader approach, through which cellular functioning should be analyzed in multiple ways, was the precursor thought to systems biology. Studies began to seek expanded roles for neurotrophic factors, not only in the peripheral and central nervous systems, but also in the immune and endocrine systems [5].

## A Century-Long Legacy

**Fig. 2:**
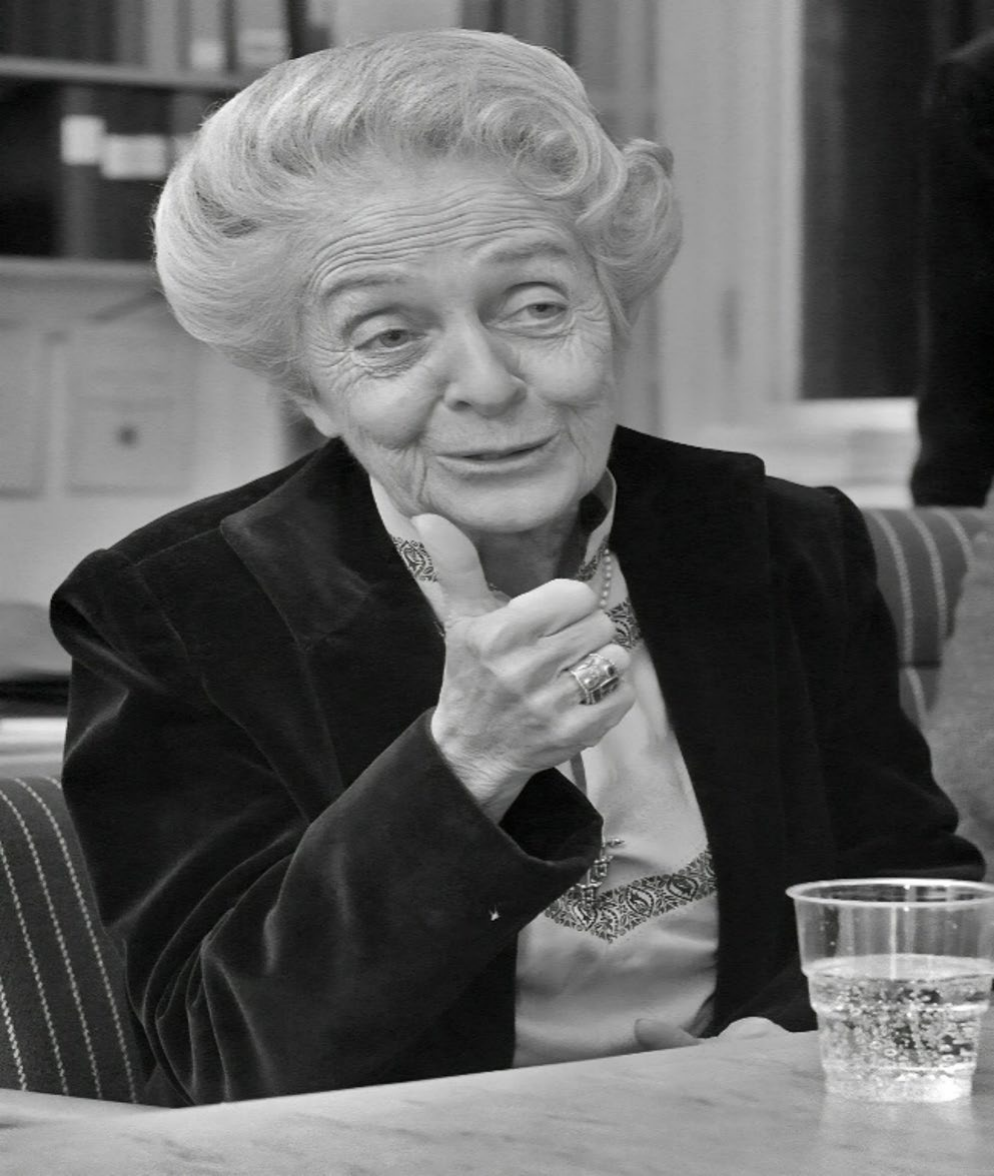
Rita Levi-Montalcini during a visit to Lund, Sweden, in 1986. Source: University Library, Lund University. Author: Kurt Hagblom, Firma Hagblom-Foto, restored by Adam Cuerden. Public Domain

Rita Levi-Montalcini (Fig. 2) was a singular and multifaceted scientist: she developed the in vitro assay for the discovery of a protein that regulates tissue growth, NGF; she discovered, with Stanley Cohen, NGF in snake venom, leading to its purification and characterization as a protein; she assumed leadership positions at EBRI [5], was awarded the Nobel Prize, in addition to having received honorary doctorate degrees from various institutions, such as the University of Uppsala in Sweden, the Weizmann Institute in Israel, McGill University in Canada, the Complutense University of Madrid in Spain, among others — significant achievements while overcoming obstacles due to sexism and religious discrimination. She was also honored with the International Saint-Vincent Prize, the Feltrinelli Prize, and the Albert Lasker Award for Basic Medical Research [70]. In 1968, she became the tenth woman elected to the United States National Academy of Sciences [71]. In 1974, she was elected a Member of the European Molecular Biology Organization [72]. Appointed Senator for Life of the Italian Republic on August 1, 2001, directly by President Carlo Azeglio Ciampi [73], she was the second woman to hold this position, after Camilla Ravera. In 2009, Levi-Montalcini became the first Nobel laureate to reach 100 years of age, in addition to being the oldest serving senator for life in the history of the Italian Republic.

In a controversial episode during cooperation with Fidia, an Italian pharmaceutical company, Levi-Montalcini supported the drug Cronassial [74], produced from gangliosides extracted from bovine nerve tissue, which was believed to favor the repair of damaged nerves, with indications for neurological diseases such as neuropathies, ataxias, neuralgias, and optic nerve atrophy [75–77]. Studies showed that the drug could be useful in the treatment of peripheral neuropathies [78–80], but over the course of treatment, some patients began to present a severe neurological syndrome, Guillain-Barré Syndrome, in which an individual’s immune system attacks their own peripheral nervous system via gangliosides. A direct and proven causal relationship was not established; the theoretical possibility of an increased risk for developing the syndrome — the drug Cronassial and Guillain-Barré Syndrome are conceptually linked by the ganglioside molecule: one used it as a treatment, the other has it as a target of the immune attack, and the safety concern, combined with the lack of robust clinical efficacy [75, 81]— led to the drug’s marketing being banned by several countries for safety reasons. In Italy, this ban came in 1993 [82].

**Fig. 3:**
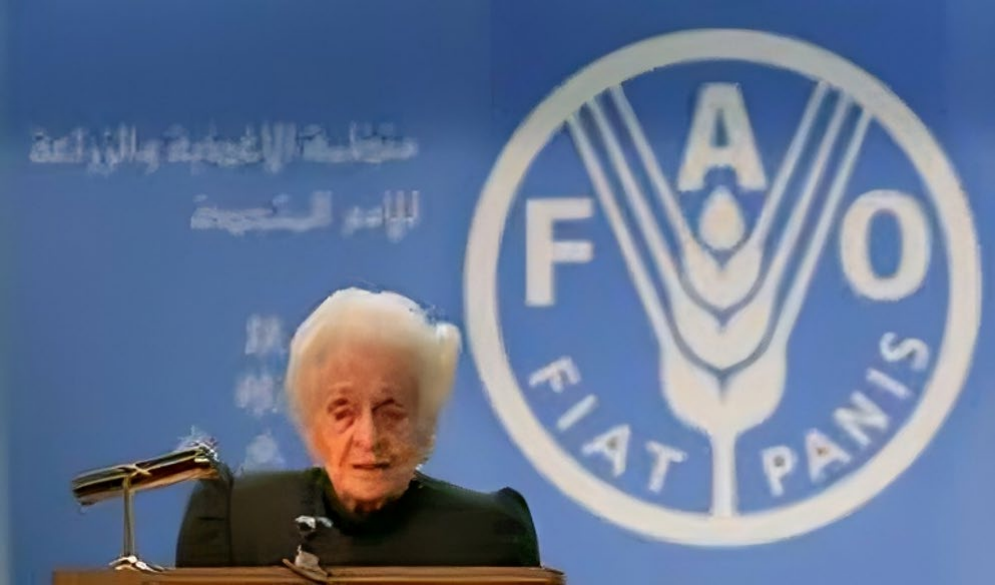
Levi-Montalcini served as an FAO Goodwill Ambassador from 1999 to 2009, when she turned 100 years old. Source: Food and Agriculture Organization of the United Nations. Reproduced with permission.

Levi-Montalcini was concerned with social issues and defended civil rights. She served as a goodwill ambassador for the Food and Agriculture Organization (FAO) **(**Fig. 3**)** of the United Nations, the agency leading efforts to eradicate hunger and combat poverty. While director of the Institute of Cell Biology in Rome, she kept a poster of Martin Luther King Jr. near her office, bearing the declaration: “A man who is not ready to die for his ideas is not fit to live”[5]. She believed that every human being’s mission is to “send a message,” and that hers was to be a good scientist and citizen, presuming that every person could contribute, provided they lived a decent life. For this reason, she reported living harmoniously without having children, science fulfilled her [7]. Her progress always depended on commitment, discipline, and patience. Influenced by Albert Schweitzer’s work in Africa, she established the Rita Levi-Montalcini Foundation to support the education and research of young women, both in Africa and Italy, through scholarships [5].

**Fig. 4:**
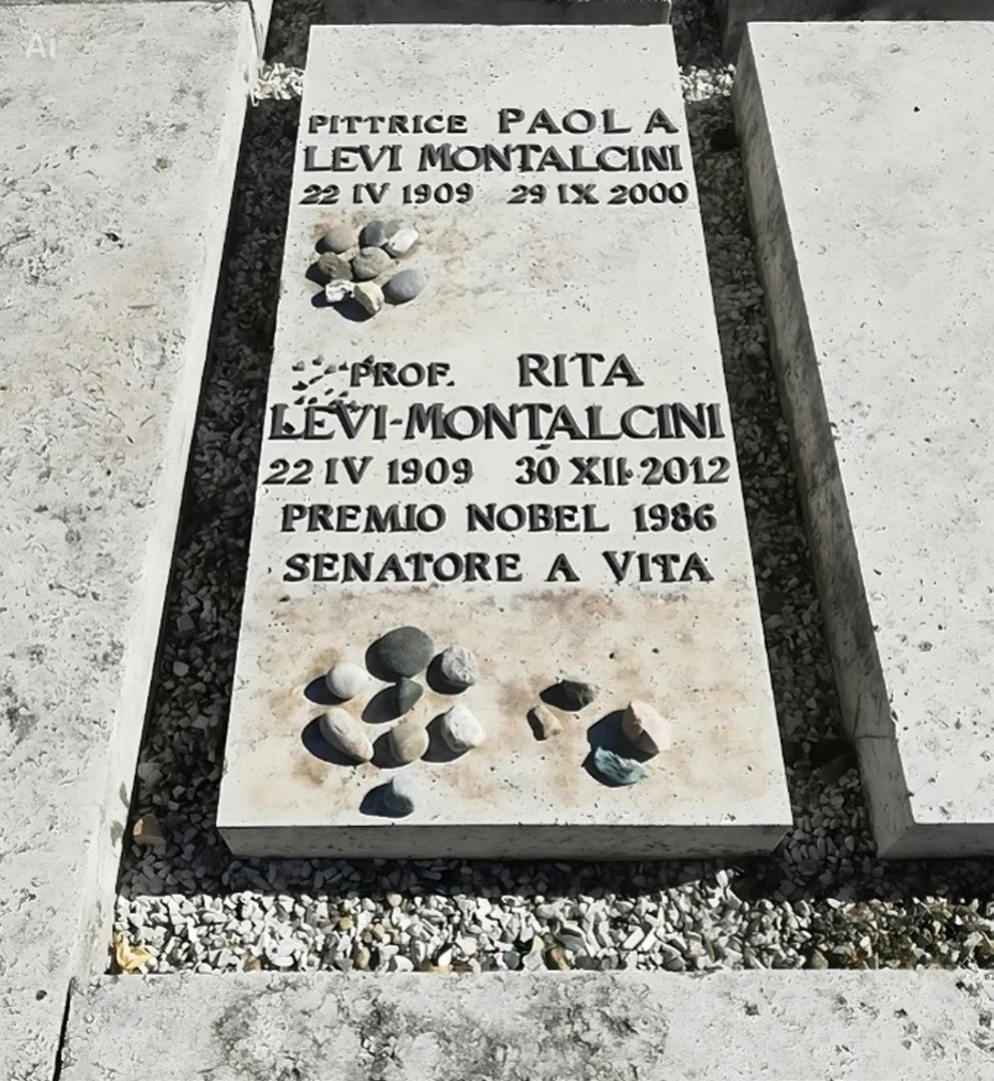
Photo of the tombstone of sisters Paola and Rita Levi-Montalcini at the Monumental Cemetery of Turin, taken during the European Day of Jewish Culture 2022. Author: Mastrocom. Creative Commons Attribution-Share Alike 4.0 International license

During her last eight years of life, she dedicated much of her time to supporting the EBRI and young scientists. She remained active in science and public life until her final years, attending the opening session of the newly elected Senate at age 97, always with strength and vulnerability coexisting. Levi-Montalcini’s story humanizes the collective tissue of science with a real narrative involving dedication, conflicts, perseverance, advances, and inevitably, mistakes. Continuing to conduct research, at 102 she published a study on how NGF could regulate axial rotation in the early chicken embryo [83], as well as a review on the function of chromaffin cells [84]. Rita Levi-Montalcini passed away at her home in Rome on December 30, 2012, at the age of 103 (Fig. 4).

## Data Availability

No datasets were generated or analysed during the current study.
